# Therapeutic Effect and Cost-Benefit Analysis of Three Different Nutritional Schemes for Esophageal Cancer Patients in the Early Post-operative Period

**DOI:** 10.3389/fnut.2021.651596

**Published:** 2021-06-17

**Authors:** Chen Huang, Xinyu Liang, Shanshan Du, Jie He, Qian Bai, Xiaoqing Feng, Xiaoqing Liu, Xu Tian, Jian Wang

**Affiliations:** ^1^Department of Nutrition, Xinqiao Hospital of Army Medical University, Chongqing, China; ^2^Department of Orthopedics, The Second Affiliated Hospital of Chongqing Medical University, Chongqing, China; ^3^Department of Thoracic Surgery, Xinqiao Hospital of Army Medical University, Chongqing, China; ^4^Nursing Department, Universitat Rovira i Virgili, Tarragona, Spain; ^5^Department of Gastroenterology, Chongqing University Cancer Hospital, Chongqing, China

**Keywords:** esophageal cancer, jejunal nutrition, short peptide jejunal nutrition, liquid equilibrium, hospital stay, cost-benefit

## Abstract

**Objective:** To retrospectively investigate the comparative efficacy, safety and cost-benefits of three nutritional treatment schemes including short peptide jejunal nutrition (SPJN), whole protein jejunal nutrition (WPJN), and partial parenteral nutrition (PPN) in patients underwent esophagectomy for esophageal cancer in our hospital. This study was carried out in accordance with the conceptual framework of nutritional therapy in fast-track rehabilitation surgery.

**Methods:** We retrospectively reviewed 305 patients who were assigned to receive esophagectomy for esophageal cancer. Eligible patients was naturally divided into SPJN group [*n* = 98 (32.1%)], WPJN group [*n* = 95 (31.1%)], and PPN group [*n* = 112 (36.7%)] according to the type of nutritional scheme which was actually prescribed to patients by the attendingphysician in clinical practice. The differences of the serum total protein (TP), albumin (ALB), pre-albumin (PA), hemoglobin (HGB), white blood cells (WBCs), red blood cells (RBCs) and neutrophils were compared among 3 nutritional schemes groups. We also investigated the relationship of the fluid intake, urine output, gastric juice drainage volume and thoracic drainage volume among 3 nutritional groups at 3 days after surgery. Moreover, the differences of cost-benefit indexes, complications, length of hospitalization and hospital expenditure were also compared.

**Results:** The serum TP, ALB, and PA in the SPJN group were all higher than those in the WPJN and PPN groups (*p* < 0.05). The gastric juice volume of gastrointestinal decompression drainage and fluid volume of thoracic drainage in the SPJN group were all less than that in the WPJN group (*p* < 0.05). The overall hospital stay and post-operative hospital stay in the SPJN group were all shorter than that in WPJN group (*p* < 0.05). Moreover, the incidence of post-operative complications including anastomotic leakage, infection, and gastrointestinal reaction was remarkably lower in the SPJN group compared to the WPJN group (*p* < 0.05). Interesting, hospital expenditure in the PPN group was less than that in the SPJN and the WPJN groups (*p* < 0.001).

**Conclusion:** Patients may obtain benefits in improving protein level after receiving SPJN scheme at the early stage after esophagectomy. Meanwhile, patients may obtain benefits in improving post-operative complications and hospital stay after receiving SPJN or PPN compared to WPJN protocol. However, the difference between SPJN and PPN requires further study because no difference was detected in terms of clinical outcomes including complications and the length of hospitalization although PPN may achieve a possible decrease of medical expenditure.

## Introduction

Issued statistics suggested that the estimated new cases of esophageal cancer are appropriate 258,000, and the cases of death are appropriate 193,000, ranking sixth in morbidity and fourth in mortality in China. The incidence of esophageal cancer in rural areas is higher than that in urban areas, and higher incidence is reported in male populations (1). According to the statistics released by the World Health Organization (WHO), morbidity and mortality of esophageal cancer in China is ranking in fifth from global perspective, and new cases and deaths in China account for about 55% of those in the world (2). Moreover, the 5-year survival rate of esophageal cancer patients in China is about 30%, which is lower than that in South Korea and Japan (3). Currently, esophagectomy is still the most common radical treatment for patients with esophageal cancer when patients experienced typical symptoms such as dysphagia and eating obstruction. Although advancements in diagnosis and treatment modalities for esophageal cancer, several aspects such as longer surgery time, a larger surgical range, higher catabolism and a longer fasting time after esophagectomy considerably increase the risk of post-operative complications such as anastomotic leakage and infection. Therefore, reasonable and standardized nutritional treatment after esophagectomy remains a critically important step for accelerating the rehabilitation of esophageal cancer patients underwent esophagectomy (3, 4).

Based on the conceptual framework of fast-track rehabilitation surgery, perioperative nutritional therapy is a critical one of the three essential treatment schedules to accelerate the recovery of patients after receiving surgery. However, most thoracic surgeons are more focused on clinical tasks, and they pay less attention to perioperative nutritional therapy and internal homeostasis for esophageal cancer patients. This in turn triggers disputes about the selection of nutritional treatment schemes during the process of clinical diagnosis and treatment. Although several studies have investigated the direct effectst and possible mechanisms of different nutritional schemes among esophageal cancer patients receiving esophagectomy, no conclusive recommendation for appropriately select nutritional scheme and delivery route, and determining the time of initiating the nutrition therapy has been generated (5, 6). More importantly, there are scant studies have been currently published to evaluate the cost-benefit of different nutritional schemes. Therefore, in this study, three different nutritional schemes including short peptide jejunal nutrition (SPJN), whole protein jejunal nutrition (WPJN), and partial parenteral nutrition (PPN), which were all initiated at the early post-operative period, were retrospectively investigated in our hospital to investigate their therapeutic effects and cost-benefit for the purpose of providing recommendations for performing perioperative nutritional therapies among patients undergoing esophagectomy for esophageal cancer.

## Materials and Methods

### Demographic and Clinical Data

Demographic and clinical data from all eligible patients who were hospitalized in the Department of Thoracic Surgery of Xinqiao Hospital in the Second Affiliated Hospital of the Third Military Medical University and were assigned to receive esophagectomy for esophageal cancer between January 2016 and December 2019 were retrospectively collected and then analyzed. The aim of the current study was to investigate the therapeutic effects and cost-benefits of three different perioperative nutritional schemes among those esophageal cancer patients underwent esophagectomy.

Inclusion criteria: (1) patient's age was 18 between and 90 years old; (2) diagnosis and treatment was made according to the *Clinical Practice Guidelines for the Diagnosis and Treatment of Esophageal Cancer* (7); (3) patients who were pathologically diagnosed with esophageal cancer were assigned to receive esophagectomy; and (4) patients signed the informed consent. Exclusion criteria: patients were (1) pregnant; (2) without sufficient targeted data; (3) with severe liver and kidney dysfunction or severe metabolic disorders; and (4) did not receive treatment for esophageal cancer. This study was approved by the Ethics Committee of the Hospital with an unique identifier of 2020-R032-01.

### Research Methods

**Figure 1:**
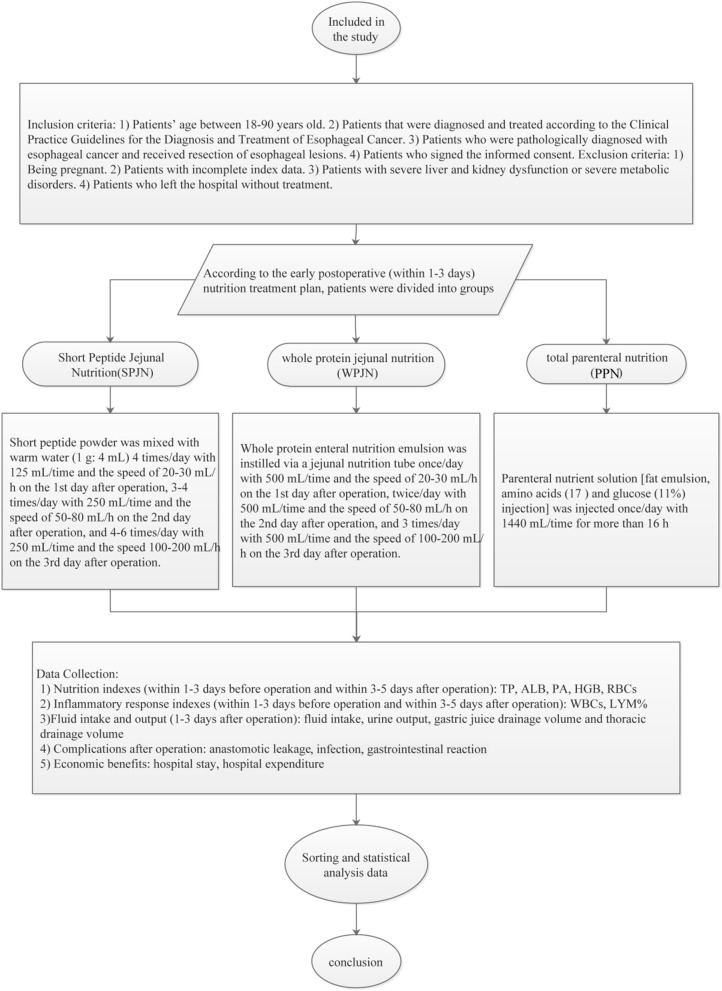
Research technology roadmap. Eligible patients were screened out using the Comprehensive Data Retrieval of Medical Care in the hospital electronic medical record system. They were divided into SPJN group, WPJN group and PPN group according to the nutritional therapy scheme in the early post-operative period (within 1–3 days). The differences in nutrition index, the inflammatory reaction index, fluid balance index, post-operative complications, cost-effectiveness and other indexes among the three groups were compared, and the transfer was analyzed and discussed. Patients benefit most from early post-operative nutrition therapy.

The eligible patients were screened out using the Comprehensive Data Retrieval of Medical Care in the hospital electronic medical record system, and then patients were naturally divided into 3 nutritional groups according to the types of nutritional schemes which were actually prescribed to patients by the attending physician in our hospital: SPJN group, WPJN group, and PPN group according to the nutritional schemes at the early post-operative period (within 1–3 days). SPJN or WPJN regime was prescribed if patients were confirmed to be tolerable to the enteral feeding or enteral feeding was not contraindication for these patients. If the nutritional goal cannot be achieved through tube feeding, parenteral nutrition route type was also applied in these patients, which was defined as PPN. In the SPJN group, the short peptide nutritional preparation (manufacturer: Nantong Richen Bioengineering Co., Ltd., product name: Revilife^®^ Short Peptide Nutrition, license No.: SC20132067100019, specification: 400 g/tin) was delivered through a jejunal tube. Specifically, short peptide powder was firstly dissolved in warm water (1 g: 4 mL) and was then digested by patients at the rate of 20–30 mL/h, 125 mL/time, 4 times/day at the 1st post-operative day, the rate of 50–80 mL/h, 250 mL/time, 3–4 times/day at the 2nd post-operative day, and the rate 100–200 mL/h, 250 mL/time, 4–6 times/day at the 3rd post-operative day. In the WPJN group, enteral nutrition emulsion (TP) [manufacturer: Fresenius Kabi Deutschland GmbH, product name: Fresubin^®^ Enteral Nutrition Emulsion (TP), Approval No.: NMPN J20140075, specification: 500 mL] was delivered via a jejunal tube at the infusion rate of 20–30 mL/h, 500 mL/time, once daily at the 1st post-operative day, the rate of 50–80 mL/h, 500 mL/time, twice daily at the 2nd post-operative day, and the rate of 100–200 mL/h, 500 mL/time, 3 times/day with at the 3rd post-operative day. In the PPN group, parenteral nutrition solution [manufacturer: Fresenius Kabi AB, product name: Kabiven^®^ Fat Emulsion, Amino Acids (17) and Glucose (11%) Injection, Approval No.: NMPN J20130185, specification: 1440 mL] was infused via the peripheral venous access with a maximum infusion rate of 150 mL/h. Specifically, fat emulsion, amino acids (17%) and glucose (11%) injection was injected once daily at 1440 mL/time within 16 h. The detailed properties and characteristics, the primary advantages and disadvantages of each nutrition scheme are shown in Table 1. The daily target energy and protein requirements for each patient within 1 week after surgery were defined to be 20–25 kcal/kg·d and 1.0–1.2 g/kg·d according to the recommendations listed in the fast-track rehabilitation surgery protocol. Other symptomatic treatments such as anti-inflammation, fluid replacement and electrolyte balance were relatively matched in the three groups of patients. Subsequently, the g demographic and clinical data of these patients were collected, and the percentages of serum total protein (TP), albumin (ALB), pre-albumin (PA), hemoglobin (HGB), white blood cells (WBCs), red blood cells (RBCs) and neutrophils within 1–3 days before surgery and within 3–5 days after surgery were compared among the three groups of patients. Concurrently, the differences in fluid intake, urine output, gastric juice drainage volume and thoracic drainage volume after operation as well as cost-benefit indexes such as post-operative complications [anastomotic leakage, infection (it was checked again by the clinician combined with the previous test results), gastrointestinal reaction including abdominal distension, abdominal pain, diarrhea, nausea and vomiting], hospital stay and hospital expenditure (all medical expenses during the hospitalization of the patients, including financial pooling for medical insurance and their own expenses for charged items) were analyzed (Figure 1).

**Table 1:** The detailed properties, characteristics and primary advantages and disadvantages of three schemes.

**Group**	**Preparation type**	**Detailed properties (/100 ml)**				**Preparation characteristics**	**Advantages**	**Disadvantages**
		**Energy (kcal)**	**Protein (g)**	**Fat (g)**	**CH (g)**			
SPJN	SPNP	82.8	3	1.34	14.46	Predigested short peptide, low fat, independent of protease and trypsin digestion and absorption	Light digestive load, good absorption, good tolerance of jejunal feeding, nutrition improvement effect is good	High osmotic pressure, the cost is higher
WPJN	ENE	100	3.8	3.4	13.8	Macromolecular protein, Normal proportion fat must rely on digestive enzymes for better digestion and absorption	Cheap and low osmotic pressure	Heavy digestive load, poor tolerance of jejunal feeding, poor nutritional improvement
PPN	PNS	69.4	2.36	3.54	6.74	Parenteral nutrition formula, high fat ratio	Easy to implement, abundant supply of energy and protein	Parenteral nutrition has heavy cardiac load, more metabolic complications, and delayed recovery of gastrointestinal function

*SPJN, short peptide jejunal nutritio; WPJN, whole protein jejunal nutrition; PPN, partial parenteral nutrition; SPNP, Short peptide nutritional preparation; ENE, Enteral nutrition emulsion; PNS, parenteral nutrient solution; CH, carbohydrate*.

### Statistical Analysis

Patients' demographic information including age, gender, and body mass index (BMI) was retrospectively collected from electronic medical system. Demographic and biochemical data were summarized as means and standard difference for the continuous variables and percentages for the categorical variable. One-way analysis of variance (ANOVA) testing was used to compare the 3 groups for continuous variables whenever data were normally distributed [expressed by (*x* ± s)]. If data we not normally distributed [expressed by M *(Q*_1_*-Q*_3_*)*], than the 3 groups were compared via rank sum test. The chi-square test was employed for the categorical variables. *P* < 0.05 indicated a statistically significant difference. All statistical analyses in this study were performed by use of Social Science Statistics version 18.0 (SPSS, IBM, Armonk, NY).

## Results

### Patient Enrollment

A total of 305 eligible patients aged 40–78 (60.7 ± 7.9) years were finally checked and then included in the study. The sample was consisted of 250 male patients (82.0%) and 55 female patients (18.0%). Among these patients, 36 patients (11.8%) experienced anastomotic leakage during hospitalization and 2 patients (0.66%) died within 1 month after discharge. There were no significant differences in the percentages of the serum TP, ALB, PA, HGB, WBCs, RBCs, neutrophils, gender, age and BMI before surgery among the three groups of patients (Table 2).

**Table 2:** Baseline data of three groups of patients.

**Index**	**SPJN (*n* = 98)**	**WPJN (*n* = 95)**	**PPN (*n* = 112)**	***P***
Age (years)	60.0 ± 8.3	61.5 ± 7.2	60.8 ± 8.0	0.419
Gender				0.078
Male	86 (87.8%)	79 (83.2%)	85 (75.9%)	
Female	12 (12.2%)	16 (16.8%)	27 (24.1%)	
BMI (kg/m^2^)	22.0 ± 2.6	22.3 ± 2.8	22.6 ± 2.8	0.317
TP (g/L)	68.2 ± 6.4	67.5 ± 6.6	67.2 ± 6.3	0.602
ALB (g/L)	43.7 ± 4.1	43.7 ± 3.7	43.8 ± 4.3	0.965
PA (mg/L)	231.8 ± 61.0	239.9 ± 57.5	221.8 ± 58.5	0.153
HGB (g/L)	134.0 ± 13.1	132.6 ± 14.8	132.3 ± 14.9	0.721
WBCs (*10^9^/L)	6.3 ± 2.9	6.4 ± 3.9	5.8 ± 1.7	0.431
RBCs (*10^12^/L)	4.3 ± 0.5	4.3 ± 0.6	4.3 ± 0.5	0.815
NEUT (%)	60.7 ± 12.8	61.4 ± 11.2	58.4 ± 12.0	0.228

*SPJN, short peptide jejunal nutrition; WPJN, whole protein jejunal nutrition; PPN, partial parenteral nutrition*.

### Nutrition Indexes

**Figure 2:**
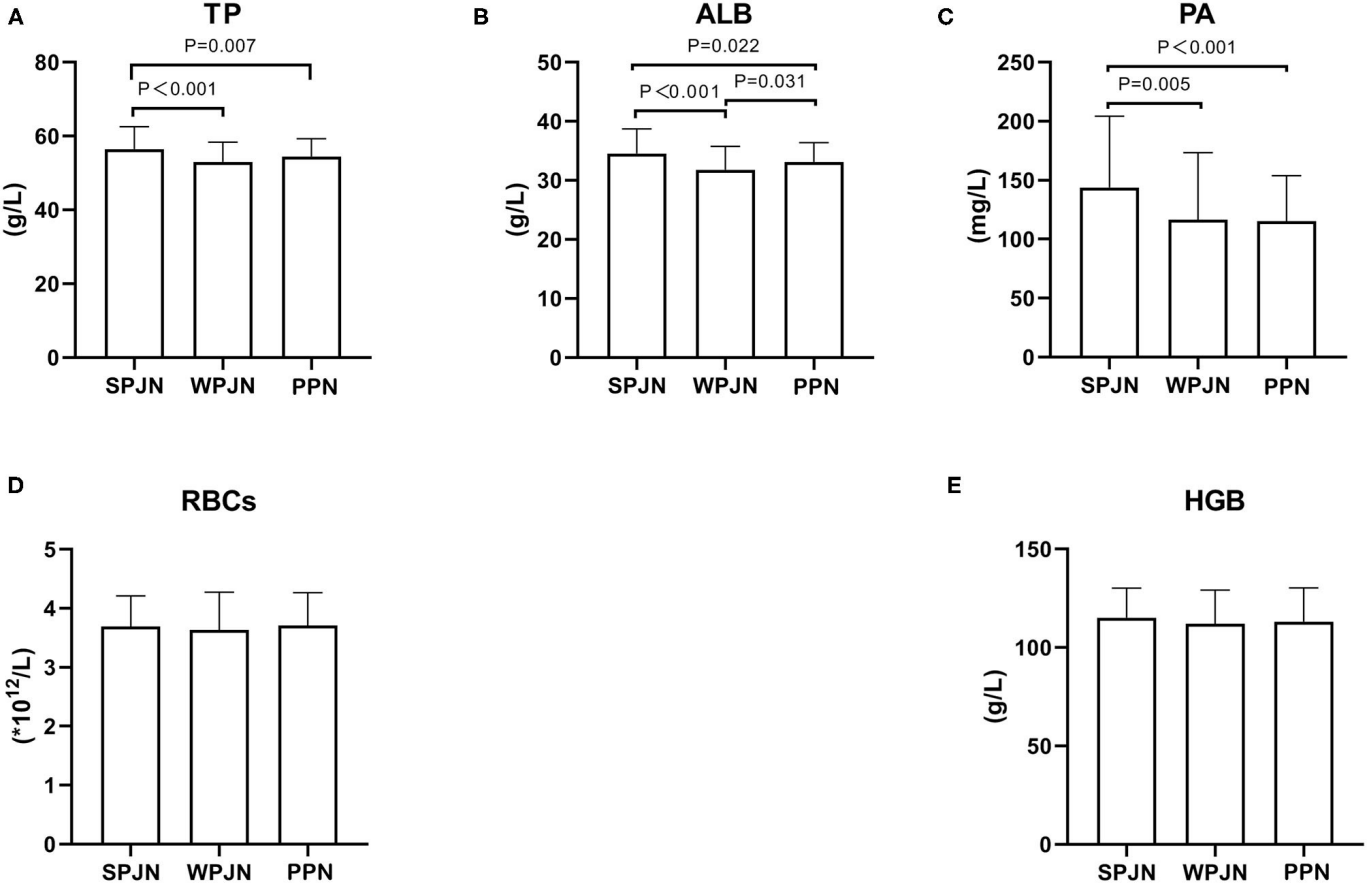
Nutrition indexes. Patients in the SPJN group had remarkably higher serum TP, ALB, PA than those in the WPJN group and in the PPN group **(A–C)**. Moreover, the ALB level after operation in the PPN group was notably higher than that in the WPJN group **(B)**. However, the RBCs and the HGB among the three groups **(D,E)** were not statistically significant.

Patients in the SPJN group had remarkably higher level of serum TP, ALB, PA than those in the WPJN [(56.50 ± 6.04) g/L vs. (53.04 ± 5.30) g/L, *p* < 0.001; (34.55 ± 4.18) g/L vs. (31.81 ± 3.97) g/L, *p* < 0.001; (143.76 ± 60.58) mg/L vs. (116.52 ± 57.02) mg/L, *p* = 0.005] and in the PPN groups [(56.50 ± 6.04) g/L vs. (54.47 ± 4.85) g/L, *p* = 0.007; (34.55 ± 4.18) g/L vs. (33.13 ± 3.25) g/L, *p* = 0.022; (143.76 ± 60.58) mg/L vs. (115.26 ± 38.66) mg/L, *p* < 0.001], seeing Figures 2A–C. Moreover, the level of ALB in the PPN group was notably higher than that in the WPJN group [(33.13 ± 3.25) g/L vs. (31.81 ± 3.97) g/L, *p* = 0.031] (Figure 2B). However, non-statistically significant differences were found in terms of RBCs [(3.69 ± 0.52) × 10^12^/L vs. (3.69 ± 0.52) × 10^12^/L vs. (3.69 ± 0.52) × 10^12^/L, *p* > 0.05] and HGB [(115.16 ± 14.99) g/L vs. (112.03 ± 17.13) g/L vs. (113.20 ± 17.06) g/L, *p* > 0.05)] among the three groups (Figures 2D,E).

### Inflammation Indexes

**Figure 3:**
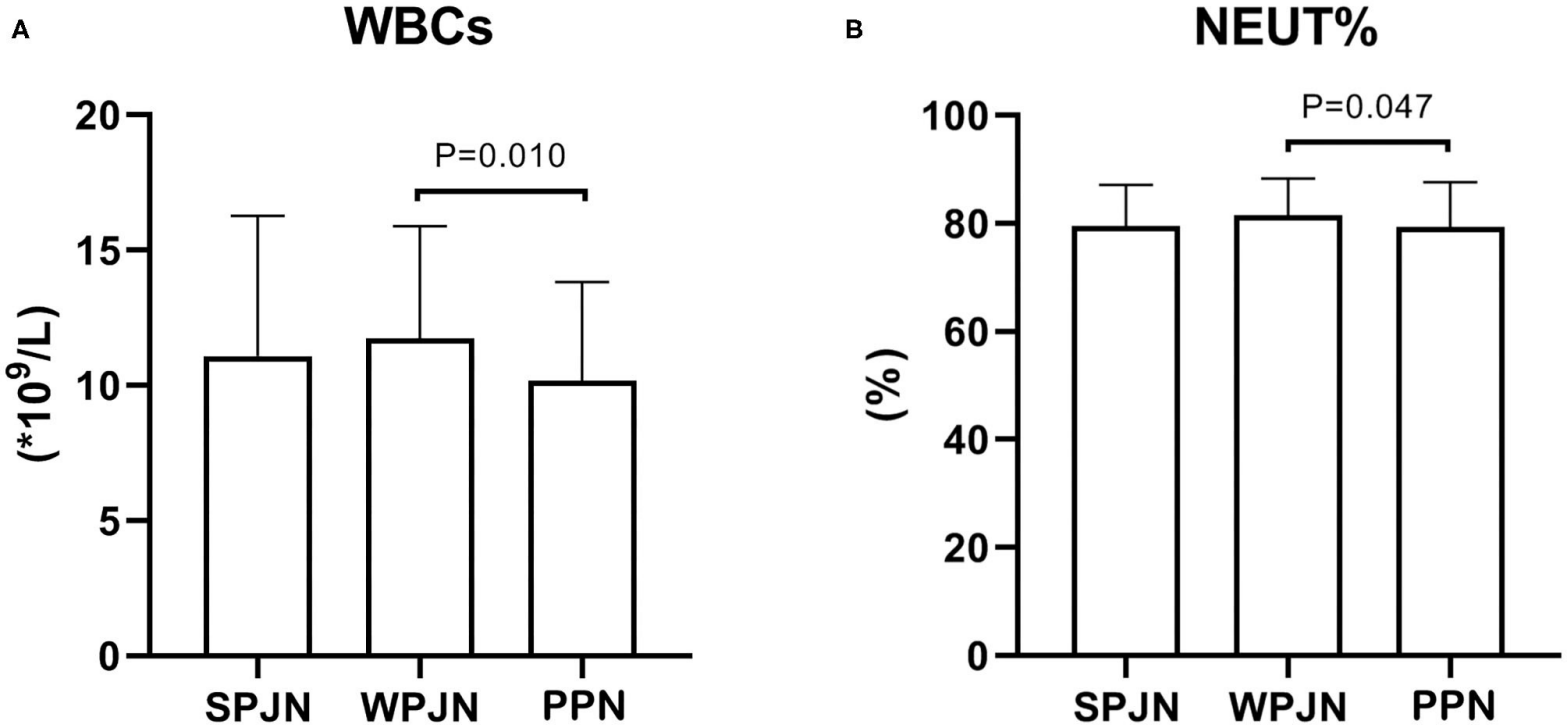
Inflammatory response indexes. The WBCs and lymphocyte percentage (LYM %) after operation were significantly lower in the PPN compared to the WPJN group **(A,B)**.

WBCs [(10.17 ± 3.66) × 10^9^/L vs. (11.74 ± 4.14) × 10^9^/L, *p* = 0.010] and lymphocyte percentage (LYM%) [(79.30 + 8.30) % vs. (81.44 ± 6.88) %, *p* = 0.047)] after surgery were significantly lower in the PPN compared to the WPJN group (Figures 3A,B). No significantly statistical difference in terms of WBCs was detected between SPJN [(11.09 ± 5.19) × 10^9^/L] and LYM% [(79.49 + 7.64) %] and WPJN groups as well as between SPJN and PPN groups (*p* > 0.05; Figures 3A,B).

### Fluid Intake and Output

**Figure 4:**
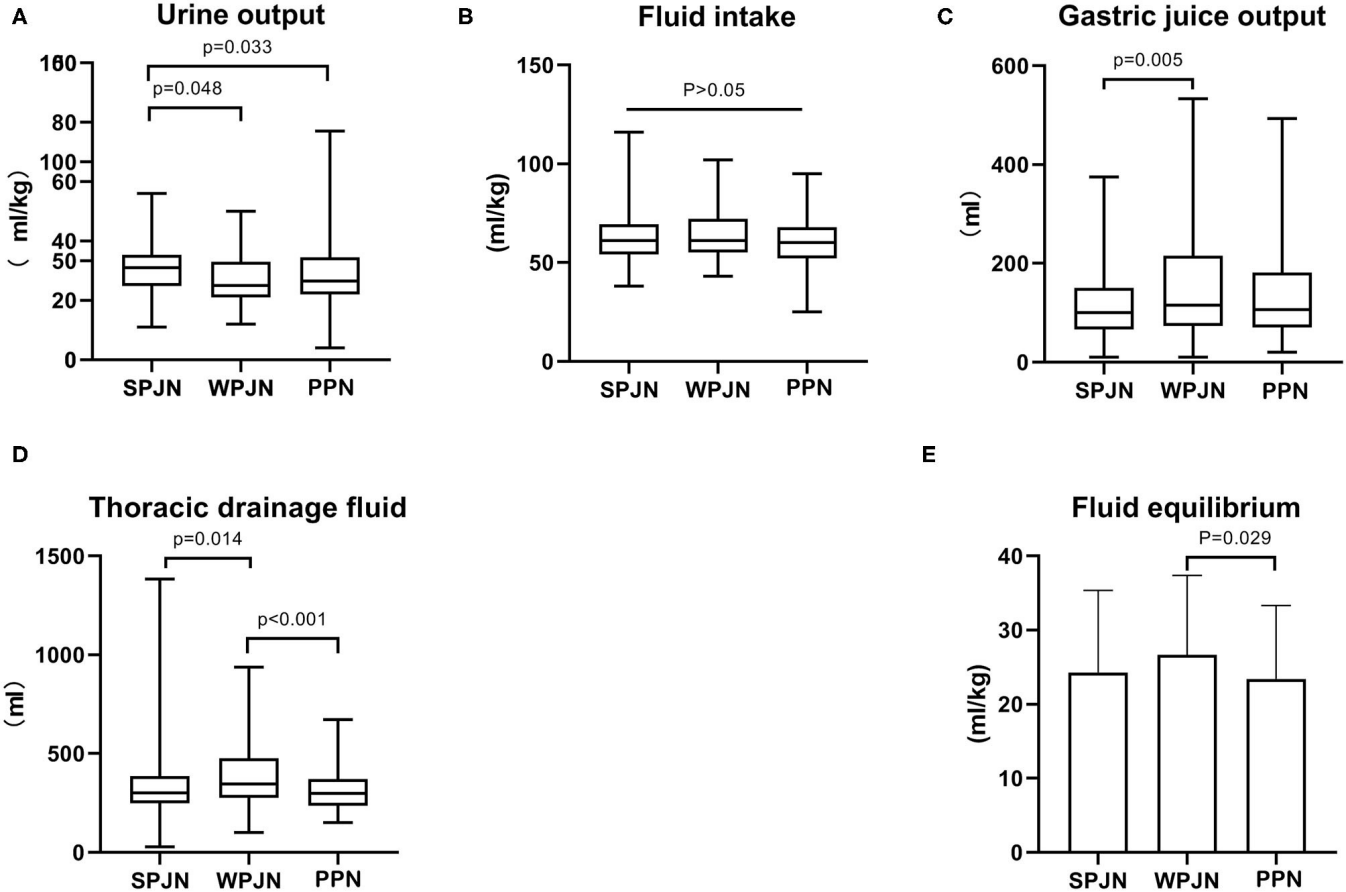
Fluid intake and output. The differences in the fluid intake among the three groups were not statistically significant **(A)**. Patients in the SPJN group had a notably larger urine output compared to patients in the WPJN group and patients in the PPN group **(B)**. Furthermore, gastrointestinal decompression drainage fluid (gastric juice output) and thoracic drainage fluid in the SPJN group were significantly lower than those in the WPJN group **(C,D)**. In addition, thoracic drainage fluid and liquid equilibrium [total fluid intake—(urine output + gastric juice output + thoracic drainage fluid)] in the PPN group were prominently lower than those in the WPJN group **(D,E)**.

Patients in the SPJN group had a notably larger urine output compared to patients in the WPJN group [31.00 (24.75–35.25) mL/kg vs. 25.00 (21.00–33.00) mL/kg, z = −1.975, *p* = 0.048) and patients in the PPN group [31.00 (24.75–35.25) mL/kg vs. 26.50 (22.00–34.50) mL/kg, z = −2.133, *p* = 0.033] (Figure 4A). The differences in the fluid intake among the three groups [61.00 (54.00–69.25) mL/kg vs. 61.00 (55.00–72.00) mL/kg vs. 60.00 (52.00–67.75) mL/kg), *p* > 0.05] were not statistically significant (Figure 4B). Furthermore, fluid volume of gastrointestinal decompression drainage (also named as gastric juice output) and fluid volume of thoracic drainage in the SPJN group was significantly lower than those in the WPJN group [100.0 (66.25–150.0) mL vs. 115.0 (73.33–215.0) mL, z = −2.778, *p* = 0.005, 300.0 (249.2–386.7) mL vs. 345.0 (276.7–475.0) mL, z = −2.468, *p* = 0.014] (Figures 4C,D). In addition, fluid volume of thoracic drainage and liquid equilibrium [total fluid intake—(urine output + gastric juice output + thoracic drainage fluid)] in the PPN group were prominently lower than those in the WPJN group [298.3 (236.7–370.0) mL vs. 345.0 (276.7–475.0) mL, z = −3.560, *p* < 0.001, (23.43 ± 9.88) mL/kg vs. (26.66 ± 10.74) mL/kg, *p* = 0.029] (Figures 4D,E).

### Post-operative Complications

**Figure 5:**
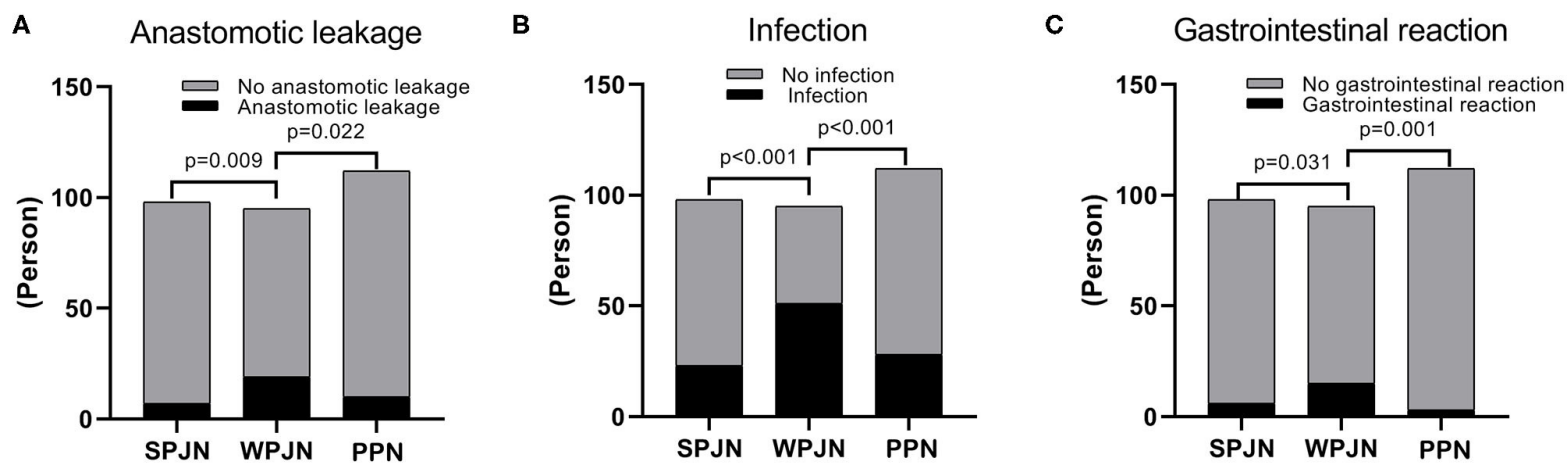
Complications. The incidence rate of anastomotic leakage in the SPJN group was significantly lower than that in the WPJN group, and it was also markedly lower in the PPN group compared to that in the WPJN group **(A–C)**.

In SPJN group, 7 patients experienced anastomotic leakage (7.14%), 23 patients were confirmed to experience infection (23.47%), 6 patients reported gastrointestinal reaction (6.12%), including diarrhea (5 cases, 5.10%) and nausea and vomiting (1 case, 1.02%). In the WPJN group, 19 patients experienced anastomotic leakage (20.00%), including 2 cases of death (2.11%); 51 patients were confirmed to experience infection (53.68%), 15 patients reported gastrointestinal reaction (15.79%), including diarrhea (13 cases, 13.68%), abdominal pain (1 case, 1.05%) and nausea and vomiting (1 case, 1.05%). In the PPN group, 10 patients experienced anastomotic leakage (8.93%), 28 patients were confirmed to experience infection (25%), 3 patients reported gastrointestinal reaction (2.68%), including diarrhea (2 cases, 1.79%) and nausea and vomiting (1 case, 0.89%). Our results revealed that the incidences of post-operative anastomotic leakage, infection and gastrointestinal reactions in the SPJN and the PPN groups were all significantly lower than those in the WPJN group (*p* < 0.05; Figures 5A–C); however no statistically significant difference was detected between SPJN and PPN groups in terms of post-operative complications (Figures 5A–C).

### Economic Benefits

**Figure 6:**
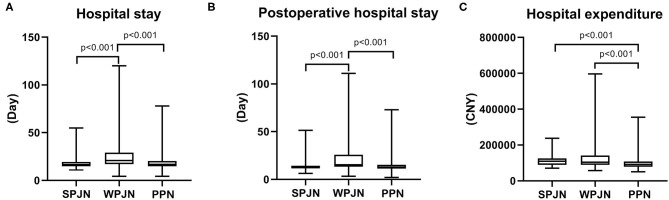
Economic benefits. Hospital stay and post-operative hospital stay in the SPJN group were remarkably shorter than those in the WPJN group, and they were significantly shortened in the PPN group compared with those in the WPJN group **(A,B)**. Moreover, patients in the PPN group spent significantly less money on hospitalization than those in the SPJN group and the WPJN group **(C)**.

Overall hospital stay and post-operative hospital stay in the SPJN [16.71 (14.97–19.35) d vs. 20.98 (17.03–29.00) d, *z* = −5.194, *p* < 0.001, 13.20 (11.39–14.37) d vs. 15.47 (13.34–25.89) d, *z* = −5.392, *p* < 0.001] and PPN [16.99 (14.98–20.50) d vs. 20.98 (17.03–29.00) d, *z* = −4.634, *p* < 0.001, 12.83 (11.35–15.49) d vs. 15.47 (13.34–25.89) d, *z* = −5.188, *p* < 0.001] groups were all remarkably shorter than those in the WPJN group (Figures 6A,B). Interesting, however, patients in the PPN group spent less medical expenditure during hospitalization than patients in the SPJN group [¥91762 (78585–107373) vs. ¥109010 (89412–125642), *p* < 0.001] and patients in the WPJN group [¥91762 (78585–107373) vs. ¥104511 (8888885–141689), *p* < 0.001] (Figure 6C).

## Discussion

### Nutritional Status

Perioperative nutritional therapy is suggested to be necessary for esophageal cancer patients undergoing esophagectomy. However, evidence suggests that the beneficial impact of nutrition treatment on patients is vary from one to another nutritional schemes. Pre-operative radiotherapy, hypoproteinemia and complications secondary to respiratory and heart diseases have been confirmed to be high risk factors for development of anastomotic leakage in esophageal cancer patients undergoing esophagectomy. Perioperative nutritional status and even subsequent nutritional therapy has great effects on the clinical prognosis and the overall survival rate of esophageal cancer patients undergoing esophagectomy. Studies suggested that rational BMI can reduce the incidence of anastomotic leakage and further improve 3-year survival rate of esophageal cancer patients receiving esophagectomy (8, 9). Excessively low prognostic nutritional index (PNI) based on serum ALB level and LYM count is an independent risk factor of affecting the overall survival rate of esophageal cancer patients (10). Perioperative nutritional treatment has a positive effect on nutritional status, and it can be delivered through PPN, nasoduodenal/nasojejunal intubation feeding, jejunostomy feeding and oral feeding. In comparison, early jejunal feeding can facilitate the recovery of gastrointestinal function, enhance immunity, increase the protein level, improve nutritional status, and decrease the risk of post-operative complications, which all in turn benefit to prognosis of patients (11–14). Some studies have also suggested that patients may also benefit from oral feeding at the early stage after esophagogastrostomy (5, 15–17). There is sparse evidence suggested that parenteral nutrition has crucial effects in supplying energy and further improving physical health at the early stage after gastrointestinal surgery (18). Both the Chinese Medical Association and the International ERAS Society made a recommendation of early jejunal nutrition support after esophagectomy (14, 19). However, most guidelines and studies only recommend the use of early jejunal nutritional support protocols, and consequently corresponding requirements have not been formulated for early enteral nutrition preparations. In this study, we investigated the comparative effects of three different nutritional schemes, i.e. SPJN, WPJN and PPN protocol, on the post-operative protein level and found that: (1) the post-operative TP, ALB and PA in the SPJN group were significantly higher than those in the PPN group (Figures 2A–C), suggesting an beneficial adjustment effect of early application of SPJN on the post-operative protein level compared to WPJN and PPN schemes; (2) the post-operative ALB in the PPN group was remarkably higher than that in the WPJN group (Figure 2B), indicating that the beneficial adjustment effect of PPN on the protein level compared to WPJN preparation.

Our analyses suggest that the reason behind our results is that the digestion and absorption of protein components in WPJN suspension can only be realized by the involvement of digestive enzymes such as gastric acid and trypsin. In China, most patients receive continuous gastrointestinal decompression lasting for 24 h after esophagectomy. limited amount of pancreatin and bile which is secreted under the stimulation of gastric juice is not sufficient to digest the macromolecular protein and fat in the jejunum because of gastric juice is sucked out via gastrointestinal decompression tube. Furthermore, evidence in China also denoted that the incidence of post-operative complications such as abdominal distension, diarrhea and abdominal pain after digesting WPJN is likely to come up with 50% (20). Meanwhile, preliminary evidence shows that patient's tolerance to enteral nutrition and overall nutritional status after digesting micromolecular SPJN or enteral nutrition which is mainly prepared based on amino acid nitrogen are notably better than patients who were instructed to digest WPJN preparation (21, 22). In this study, the levels of serum TP, ALB and PA in the SPJN group were markedly higher than those in the WPJN group, which further revealed that pre-digested SPJN preparation should be given for enteral nutrition in order to effectively improve the protein level of patients.

### Fluid Intake, Output, and Inflammatory Responses

The International ERAS Society recommends 30 mL/kg/d of fluid after esophagectomy in the *Guidelines for Perioperative Care in Esophagectomy*. Even if the blood pressure is maintained at not <80% of the normal level, the intake of fluid as low as 12 mL/kg/d will not lead to renal failure (14). Therefore, goal-oriented fluid replacement is advocated in the concept of fast-track rehabilitation surgery. Many studies have also confirmed that goal-oriented fluid replacement can alleviate inflammatory responses, facilitate the recovery of intestinal function and reduce the risk of post-operative complications (23–26). Fluid intake of all patients in this study was relatively high (median: 60–61 mL/kg/d). The fluid intake and urine output are all clinically positive adjustable factors, while fluid volumes of gastrointestinal decompression drainage and thoracic drainage is passive indexes reflected by the condition. The volume of gastrointestinal decompression drainage is linked to the secretion of gastric juice, which is secreted under the stimulation of food. The fluid volume of gastrointestinal decompression drainage in the WPJN group was the largest because its significant chemical stimulation from macromolecular proteins in WPJN preparation, while it was the smallest in the SPJN group, which was comparable to that in the PPN group (Figure 4C). It implies that admission of SPJN and PPN may contribute to reduce the secretion of gastric juice, and even exert a better effects than fasting. The fluid volume of thoracic drainag is depend upon the degree of inflammatory, blood pressure and protein level, however the degree of inflammatory is depend upon the liquid equilibrium, and thus the liquid equilibrium will critically affect the fluid volume of thoracic drainage. Excessive liquid equilibrium will increase the degree of tissue edema, and then aggregate the degree of inflammatory response, which is supported by limited fluid resuscitation and goal-directed fluid therapy. Our current study revealed that PPN keep a promising liquid equilibrium, and thus this protocol was associated with the relieved inflammatory response (Figure 4E). However, no statistical difference was detected between SPJN and PPN groups in terms of liquid equilibrium. Furthermore, WBCs and LYM% in the PPN group were evidently lower than those in the WPJN group (Figures 3A,B), and the thoracic drainage fluid in the PPN group was also the least (Figure 4D), however no statistical difference was detected between SPJN and PPN groups in terms of these three indexes. The recommendation that arises from these results is that clinically attending doctors would need to control the infusion of post-operative fluid, and evaluate the equilibrium of fluid intake and output. If the volume of therapeutic fluid exceeds the standard, diuretics should be utilized to increase urine output in time.

### Complications and Cost-Benefit

Complications and cost-benefit constitute crucial indicators in clinical treatment. All medical treatments should aim at achieving the best quality of life with the least medical cost. Due to the special physiological and anatomical location and structural characteristics of the esophagus, the incidence of complications after esophagectomy is relatively high. The most serious complication is the anastomotic leakage, which severely affects prognosis and the quality of life of patients undergoing esophagectomy. Significant advancements in medical technology, especially jejunum nutrition, have contributed to the decline of mortality caused by anastomotic leakage after esophagectomy. The incidence of anastomotic leakage after esophagectomy is at 10-20% according to published studies performed by European and American scholars (17, 27), while it is at 3–7% according to the reports of Japanese scholars (28). Due to several differences including tumor stage, indications and lymph node dissection between NCCW and JES, the United States has a large proportion of salvage surgeries for advanced esophageal cancer, which are risky and lead to a higher incidence of post-operative complications. In 2015, the Society of Thoracic Surgeons reported that complications after esophagectomy reached 62.2%, and the perioperative mortality rate was 3.8% (29). In this study, the overall incidence of anastomotic leakage was 11.6%, which was lower than that in Europe and America and comparable to the finding in Japan. The incidence of anastomotic leakage was the lowest in the SPJN group (7.4%) and the highest in the WPJN group (20%) (Figure 5A), and the mortality in the WPJN group was 2.11%. This result suggests that not all enteral nutrition can reduce post-operative complications, and nutritional therapy with WPJN protocol may aggravate unfavorable clinical outcomes of patients. Additionally, the overall hospital stay and post-operative hospital stay in the SPJN and PPN groups were all also predominantly shorter than that in the WPJN group (Figures 6A,B), however no statistical difference was detected between SPJN and PPN in terms of these two outcomes. Generally, parenteral nutrition is definitely more expensive than enteral nutrition. Interesting, however, the hospital medical expenditure in the PPN group (91762 RMB) was lower than that in SPJN (109010 RMB) and WPJN (104511 RMB) groups in the current study (Figure 6C). After deeply reviewing all outcomes, we infer that patients receiving PPN spent the lowest medical expenditure during hospitalization due to the following possible reasons: (a) PPN scheme was associated with the lowest risk of inflammatory response compared to SPJN [WBCs: (10.17 ± 3.66) ^*^10^9^/L vs. (11.09 ± 5.19) ^*^10^9^/L], and thus fewer antibiotics were prescribed in practice which is potentially associated with lower medical expenditure; (b) patients receiving PPN scheme experienced shorter post-operative length of hospitalization (13.20 days) compared to other patients receiving SPJN (12.83 days) and WPJN (15.47 days) although no statistical difference between SPJN and PPN groups was detected; (c) no jejunostomy tube, which is definitely more expensive than infusion support, will be required in patients receiving PPN; and (d) no more medical strategies were prescribed to address post-operative complications including anastomotic leakage and infection compared to patients receiving enteral nutrition including SPJN and WPJN because statistical difference was not achieved in terms of these outcomes.

## Conclusion

In conclusion, our results demonstrated a promising benefits in terms of post-operative protein level when patients were treated with SPJN preparation at the early stage after esophagectomy. Meanwhile, our results also suggested a promising benefits in terms of post-operative complications and hospital stay when patients were treated by SPJN or PPN compared to WPJN. However, the difference between SPJN and PPN requires further study because no difference was detected in terms of clinical outcomes including complications and hospitalization. Finally, this study presents some limitations in its design. These limitations include: traditional concepts in clinical treatment, review of experimental indicators, and sample size. Moreover, we did not report how much energy and protein were received within each group because of no information can be extracted from the electronic medical system, and thus future study should be performed to investigate dose effect of different nutritional protocols. The cost-benefit advantage of SPJN has not been effectively demonstrated in this study. Therefore, the authors are planning to conduct a prospective clinical controlled study in order to address above limitations in the future.

## Data Availability Statement

The raw data supporting the conclusions of this article will be made available by the authors, without undue reservation.

## Ethics Statement

The studies involving human participants were reviewed and approved by Ethics Committee of the Xinqiao Hospital of Army Medical University. The patients/participants provided their written informed consent to participate in this study.

## Author Contributions

CH, XLia, and JW conceived this study. CH, SD, QB, XF, and XLiu participated in data collection. CH, JH, and XT performed statistical analysis. CH drafted the manuscript. All authors reviewed and approved the final manuscript.

## Conflict of Interest

The authors declare that the research was conducted in the absence of any commercial or financial relationships that could be construed as a potential conflict of interest.

## Abbreviations

SPJN: short peptide jejunal nutrition
WPJN: whole protein jejunal nutrition
PPN: partial parenteral nutrition
TP: total protein
ALB: albumin
PA: prealbumin
HGB: hemoglobin
WBCs: white blood cells
RBCs: red blood cells
WHO: world health organization.
